# SOFA-2 represents meaningful progress toward a more inclusive and equitable global sepsis assessment framework

**DOI:** 10.3389/fmed.2026.1850281

**Published:** 2026-06-16

**Authors:** Carolina Hincapié-Osorno, Fabián Jaimes, Hjalmar R. Bouma

**Affiliations:** 1 Grupo de Investigación en Urgencias y Emergencias (GIURE), University of Antioquia, Medellín, Colombia; 2 Department of Internal Medicine, University Medical Center Groningen, University of Groningen, Groningen, Netherlands; 3 Department of Internal Medicine, University of Antioquia, Medellín, Colombia; 4 Department of Acute Care, University Medical Center Groningen, University of Groningen, Groningen, Netherlands; 5 Department of Clinical Pharmacy and Pharmacology, University Medical Center Groningen, University of Groningen, Groningen, Netherlands

**Keywords:** critical care, global health equity, resource-limited settings, sepsis, Sequential (Sepsis-related) Organ Failure Assessment (SOFA), SOFA-2 score

## Abstract

The updated Sequential Organ Failure Assessment (SOFA-2) score introduces important improvements in clarity, reproducibility, and global representation. By incorporating explicit scoring rules and data from a limited number of low- and middle-income countries (LMICs), it begins to address long-standing concerns regarding variability and applicability. However, substantial imbalances in data representation and health system capacity persist, and the extent to which these advances translate into improved calibration across diverse settings remains to be established. We argue that while SOFA-2 is a meaningful step forward, future sepsis frameworks must integrate context-specific evidence, account for structural constraints, and prioritize equitable participation in model development to ensure globally valid and clinically actionable tools.

## Introduction

We welcome the publication of SOFA-2 as an important update to a widely used clinical tool in the assessment of sepsis (1). The Sequential Organ Failure Assessment (SOFA) score, previously termed the Sepsis-related Organ Failure Assessment, has long reinforced sepsis research, clinical trials, and bedside decision-making, functioning both as a descriptor of illness severity and as a prognostic indicator. Its widespread adoption, however, has also exposed important limitations in consistency, interpretability, and global applicability (2). SOFA-2 represents a deliberate effort to address these shortcomings and marks a notable advance in the evolution of sepsis assessment frameworks.

### From implicit judgment to explicit rules, laying the foundation for reproducibility

Several long-standing limitations of the original SOFA are now addressed through explicit rules rather than reliance on individual clinician interpretation (1, 3). For example, patients on chronic dialysis are now assigned to a specific kidney dysfunction score rather than being scored variably depending on local practice or clinician judgment (1). Clear guidance is also provided for the handling of missing values, a frequent challenge in real-world datasets and especially in resource-limited settings. Specifically, PaO2/FiO2 ratios may be replaced with SpO2/FiO2 ratios when arterial blood gas analysis is unavailable, and absent Glasgow Coma Scale (GCS) values may be assumed normal under predefined conditions (1). These clarifications strengthen reproducibility, reduce arbitrary variability in scoring, and enhance comparability across studies and clinical environments. The emphasis on explicit rules also has important implications for research integrity. In multicenter and multinational studies, even small variations in score calculation can lead to meaningful differences in patient classification and outcome estimates. By standardizing assumptions and substitutions, SOFA-2 reduces hidden sources of bias and enhances transparency. This is particularly relevant in an era where SOFA-based definitions influence trial eligibility, benchmarking, and quality metrics. In this respect, SOFA-2 improves not only clinical usability but also methodological rigor. However, organ dysfunction scores remain downstream summaries of complex host–pathogen interactions and do not resolve the underlying biological heterogeneity may drive variation in disease trajectories and treatment response (4).

### A step toward global representation

The most consequential advance, however, is the inclusion of data from low- and middle-income countries (LMICs), notably Brazil and Nepal. This represents a meaningful departure from Sepsis-1 (5), Sepsis-2 (6), and Sepsis-3 (7), all of which were developed exclusively using data from high-income countries (HICs) (Table 1), despite LMICs bearing the overwhelming majority of global sepsis morbidity and mortality (8). This historical imbalance has long raised concerns about the external validity of sepsis definitions when applied to health systems that differ substantially in infrastructure, workforce capacity, and patient pathways. Furthermore, earlier foundational sepsis publications were not open access, including Sepsis-1 (5) and the original SOFA (3), creating a structural barrier to dissemination and uptake in precisely the settings where sepsis burden is greatest. Equity requires transparency not only in dissemination but also in authorship, data ownership, and agenda-setting. Without meaningful inclusion in the knowledge-production process, LMICs risk remaining passive recipients of frameworks that may not fully reflect their realities.

**TABLE 1:** Evolution of sepsis definitions.

Definition	Year	Key organiza-tions	Criteria
Sepsis-1	1991	ACCP, SCCM	Systemic inflammatory response syndrome (SIRS ≥ 2) to infection
Sepsis-2	2001	SCCM, ESICM, ACCP, ATS, SIS	Infection with systemic inflammation
Sepsis-3	2016	SCCM, ESICM	Infection with acute organ dysfunction (SOFA score ≥ 2)

ACCP, American College of Chest Physicians; SCCM, Society of Critical Care Medicine; ESICM, European Society of Intensive Care Medicine; ATS, American Thoracic Society; SIS, Surgical Infection Society.

**FIGURE 1:**
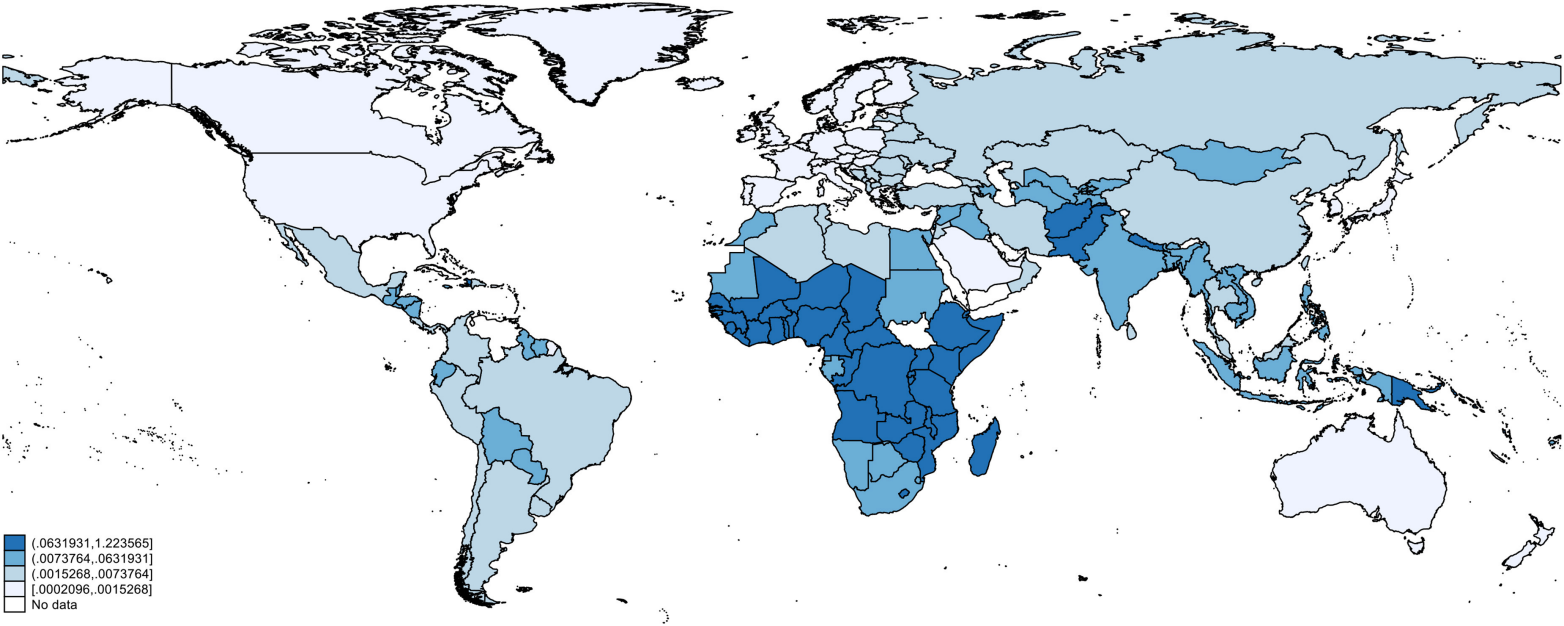
Age standardized sepsis mortality per 100,000 population in relation to GDP per capita at purchasing power parity, 2017. Map created with data from the Global Burden of Disease Study (8) and the World bank open data (12) to illustrate the persistent mismatch between sepsis burden and economic capacity.

SOFA-2’s inclusion of LMIC data signals progress, but the scale of the remaining challenge becomes visible when sepsis burden is placed in its economic context. Figure 1 maps age-standardized sepsis mortality against GDP per capita at purchasing power parity for 2017, revealing a pronounced and clinically consequential pattern: the countries bearing the highest sepsis mortality are systematically those with the lowest economic capacity to invest in the critical care infrastructure, mechanical ventilation, vasopressors, renal replacement therapy, laboratory diagnostics, and trained workforce, that underpins the development and routine application of organ dysfunction scores such as SOFA-2. This structural reality carries two direct implications for the validity of SOFA-2 across contexts. First, these populations were largely absent from the datasets in which SOFA-2 and its predecessors were developed and validated. Second, the clinical assumptions embedded in the score, including availability of organ support, monitoring frequency, and laboratory access, may not hold in precisely the settings where sepsis burden is greatest, raising substantive questions about calibration and interpretability that go beyond representational imbalance alone. Against this backdrop, it remains difficult to justify the continued underrepresentation of populations most impacted by sepsis in guideline development and validation processes.

Encouragingly, recent initiatives demonstrate that this imbalance is neither inevitable nor insurmountable. The global Delphi consensus on sepsis care in resource-limited settings (9) shows that broad, international participation is not only feasible but methodologically advantageous. By incorporating diverse clinical perspectives, such processes can identify context-specific priorities that may be invisible to experts practicing in well-resourced environments. Although SOFA-2 reflects progress in this direction, HIC perspectives still dominate the dataset (88.7%), raising concerns that implicit assumptions about care pathways, monitoring frequency, and treatment availability may persist. A Bayesian perspective further highlights this concern. Because posterior probabilities depend critically on prior likelihoods, substantial differences in baseline sepsis prevalence, delays in presentation, antimicrobial resistance patterns, and diagnostic capacity challenge the validity of uniform decision rules across settings. Models derived largely from HIC datasets are at risk of misestimating severity and misclassifying patients when applied to LMIC contexts, where patients often present later in the disease course and with fewer prior interventions though direct comparative validation studies are needed to quantify the extent of this miscalibration. These differences are not merely statistical; they directly affect triage decisions, prognostication, and resource allocation.

### When models travel, the problem of context

Bayesian reasoning underscores that clinical reasoning is probabilistic rather than absolute. Interpretations and inferences should evolve as new evidence accumulates, weighted against prior assumptions shaped by historical studies, clinical experience, cultural norms, and the structural conditions of care. A single study may be insufficient to overturn an established prior, but as evidence accumulates, conclusions should be updated accordingly. For this process to function, however, the underlying data must be both high quality and contextually relevant. The FEAST trial in sub-Saharan Africa provides a powerful illustration (10). Contrary to expectations derived from HIC-based sepsis trials, aggressive fluid resuscitation in children with severe infection was associated with increased mortality, particularly in settings without access to mechanical ventilation. One plausible explanation is that limited critical care capacity increased vulnerability to fluid overload and pulmonary edema, revealing an interaction between biological insult and system constraints rather than a contradiction of physiology. This experience underscores that evidence generated in well-resourced environments cannot be assumed to generalize universally. When such evidence is nonetheless embedded into severity scores and decision rules, miscalibration is no longer theoretical but operational.

Consider two patients each presenting with a SOFA-2 score of 8. The first is admitted to a tertiary hospital in a high-income country, where the score triggers ICU admission, vasopressor initiation, and close monitoring with repeated laboratory assessment every 6–8 h. The clinical team has access to mechanical ventilation, renal replacement therapy, and culture-directed antimicrobial therapy with rapid susceptibility results. The second patient presents to a district hospital in a low-income country, where the same SOFA-2 score of 8 cannot trigger the same response: ICU beds are unavailable, vasopressors are in limited supply, arterial blood gas analysis is not routinely possible, and the patient has already experienced a delay of 48 h between symptom onset and first clinical contact, a delay that is common in settings where access to care is constrained by distance, cost, or workforce limitations. Furthermore, the local prevalence of antimicrobial-resistant organisms is substantially higher, reducing the probability that empirical treatment will be adequate. The score is identical; the prognostic meaning and the realistic therapeutic response are not. For this second patient, a SOFA-2 score of 8 may represent a substantially higher posterior probability of a poor outcome than the derivation dataset, drawn predominantly from HIC populations, would predict. These are not exceptional scenarios: they represent the structural reality of sepsis care for the majority of patients globally.

### Beyond physiology, the role of health system capacity

Importantly, severity scores such as SOFA do not function solely as descriptive instruments; they also shape clinical behavior. In many health systems, changes in SOFA score influence decisions regarding ICU admission, escalation of antimicrobial therapy, or referral to higher-level facilities. In LMICs, however, these downstream responses are frequently constrained by bed availability, staffing shortages, limited diagnostic capacity, and transport barriers. Consequently, identical SOFA scores may carry fundamentally different clinical implications depending on context. This divergence raises critical questions about whether severity scores should be interpreted purely as biological markers of organ dysfunction or whether they should also be contextualized within the realistic therapeutic options available. Organ dysfunction scores quantify physiological derangement, but outcome is co-determined by health system capacity, making disease severity and survival probability non-equivalent constructs. Without such consideration, uniform thresholds risk identifying need without the capacity to respond, thereby reinforcing existing inequities. These challenges extend beyond individual patient care to health system evaluation and policy. SOFA-based severity adjustment is increasingly used in hospital benchmarking, outcome comparisons, and assessments of care quality. If the underlying scoring system is insufficiently validated for LMIC populations, such comparisons may inadvertently penalize under-resourced systems while obscuring structural determinants of poor outcomes. This has implications for funding allocation, public reporting, and international performance comparisons. A globally applicable SOFA framework must therefore perform robustly not only at the bedside but also in aggregate analyses that inform policy. Equity-aware validation strategies, such as stratified analyses by income level, care capacity, or geographic region, could help ensure that SOFA-2 supports accountability without perpetuating bias.

Structural constraints also raise unresolved conceptual questions. For example, how should kidney dysfunction be interpreted in environments where transplantation is unavailable and dialysis represents the only form of renal replacement therapy? Similarly, how should neurologic scores be contextualized where sedative use, monitoring frequency, or staffing ratios differ markedly from those assumed during model development? Without explicit attention to these realities, even well-calibrated scores may produce misleading signals when transported across health systems with fundamentally different constraints. At the same time, we commend the developers of SOFA-2 for retaining a pragmatic, clinically grounded approach that avoids reliance on high-cost or technologically intensive tools. By prioritizing variables that are routinely available in most hospital settings, the score maintains feasibility across a wide range of resource environments. This design choice increases SOFA-2’s potential for universal use and represents a meaningful step toward more equitable tools in global sepsis care. Importantly, it also acknowledges that incremental improvements in applicability may ultimately be more impactful than theoretically optimal but impractical models.

## Discussion

### Toward equitable and context-aware sepsis frameworks

SOFA-2 represents a welcome and meaningful advancement in global sepsis assessment. Its value lies not in having resolved the equity challenges of previous frameworks, but in having moved the field in the right direction: by standardizing scoring rules, improving reproducibility, and for the first time, incorporating data from LMIC settings and making the publication open access. These are meaningful steps precisely because they create a foundation that did not previously exist. The limits of that progress are, however, equally important to acknowledge. With 88.7% of development data originating from high-income settings (1), SOFA-2 remains a framework derived predominantly from a context that represents a minority of the global sepsis burden. Whether its performance is equivalent across the full spectrum of health system environments is a question that has not yet been directly answered empirically, and one that the field must now prioritize.

Future frameworks should also consider integration with biologically informed subtyping approaches, as uniform thresholds may obscure clinically relevant heterogeneity in host response. Importantly, as the field moves toward a potential Sepsis-4 consensus, there is now a valuable opportunity to build on this foundation by fully integrating stakeholders from LMICs from the outset rather than relegating their role to *post hoc* validation (11). This requires a specific and operational research agenda. First, multicountry cohorts drawing on LMIC populations across multiple geographic regions, not limited to Brazil and Nepal, should be prospectively established to participate in both the development and validation of future sepsis frameworks, ensuring that LMIC clinical realities shape the model from the outset rather than serving only as a *post hoc* test of tools derived elsewhere. Second, context-specific validation/calibration studies should test SOFA-2 performance under different ICU admission thresholds, resource-availability scenarios, and antimicrobial resistance profiles. Third, all validation studies should transparently report the frequency and pattern of missing physiological and laboratory data, as data completeness is itself a health system variable that affects score performance. Fourth, participatory governance structures should give LMIC researchers meaningful agency in agenda-setting and model development, rather than data-provision roles. Fifth, sensitivity analyses stratified by income level and health system capacity should be pre-specified in future validation protocols to ensure that aggregate performance estimates do not mask subgroup miscalibration. Only through such inclusive and collaborative approaches can sepsis definitions truly “go far,” aligning methodological rigor with global equity and ensuring that the tools designed to measure sepsis reflect the populations most affected by it.

## Data Availability

The original contributions presented in this study are included in this article/supplementary material, further inquiries can be directed to the corresponding author.
